# Geographic range size and rarity of epiphytic flowering plants

**DOI:** 10.1038/s41477-025-02022-9

**Published:** 2025-06-13

**Authors:** Vida J. Svahnström, Eimear Nic Lughadha, Félix Forest, Tarciso C. C. Leão

**Affiliations:** 1 Royal Botanic Gardens, Kew, Richmond, UK; 2 Department of Life Sciences, Imperial College London, Silwood Park, Ascot, UK; 3 Science and Solutions for a Changing Planet DTP, The Grantham Institute, Imperial College London, South Kensington, London, UK

**Keywords:** Biogeography, Conservation biology, Plant ecology

## Abstract

For over a century, epiphytes have been considered to have larger geographic ranges than terrestrial plants, yet this assumption is based on studies at restricted geographic and taxonomic scales and is contradicted by recent research. Misunderstanding the ranges of epiphytes may distort perceptions of their extinction risk. To address this, here we analysed global data on 330,087 angiosperm species, including 27,184 epiphytes, comparing range size and rarity between epiphytes and terrestrial plants. We calculated three range metrics, tested for differences across angiosperms and within epiphyte-rich families and used phylogenetic regressions to explore the role of epiphytism on species ranges. On average, epiphytes have larger ranges than closely related terrestrial species, supporting hypotheses that epiphytism promotes dispersal. However, small ranges are prevalent in epiphyte-rich families regardless of lifeform. Notably, about half of epiphyte species are rare, indicating greater vulnerability than terrestrials. Epiphyte rarity is attributable to evolutionary history and shared traits rather than epiphytism itself.

## Abstract (web summary)

Epiphytes have larger ranges than closely related terrestrial species. However, a large proportion of species in epiphyte-rich clades are rare with small range sizes, regardless of their lifeform.

## Main

The area over which species are distributed, their geographic range size, is a key factor underpinning global patterns of plant diversity and vulnerability to extinction^1,2^. Range size is a major axis of rarity^3,4^. Despite the lack of a universal definition, rare species are typically considered to be those that have restricted distributions, low abundance or both^3^. Rare species are more likely to be threatened with extinction than common and widespread species^1^; four of the five criteria used to categorize species as threatened on the International Union for Conservation of Nature (IUCN) Red List of Threatened Species consider either absolute levels or trends in species abundances or distributions^5^. Range size is the most frequently used metric for assessing threatened plant species on the Red List^6^ and it is probably the single most important indicator of the global extinction risk of a species. The smaller a species range size is, the higher the probability that environmental changes or habitat loss may affect its entire population and eventually lead to extinction^1^. Thus, understanding drivers and correlates of rarity, and range size in particular, will enhance our ability to predict which species are at risk of extinction.

Epiphytes, plants that germinate and root non-parasitically on other plants at all life stages^7^, comprise ~8% of angiosperm species globally^8^, ≤20–39% of vascular flora in tropical plant diversity centres^9^ and >50% of plant species in local inventories in certain tropical regions^10,11^. Epiphytes provide critical arboreal habitats^12,13^ and contribute to water and nutrient cycles in tropical forests^14^. Among angiosperms, epiphytism occurs in nearly 60 families, but most epiphyte diversity is concentrated in very few species-rich lineages; orchids alone comprise 75% of angiosperm epiphyte species^8^. Highly dependent on their hosts and the availability of atmospheric moisture, angiosperm epiphytes are considered particularly susceptible to the effects of land use and climate changes in the tropics^15^. Understanding the relationship between epiphytism and range size provides insight into the vulnerability of epiphytes to such threats.

That epiphytes generally have larger ranges than terrestrial plants has been widely accepted since Schimper (1888)^16,17^. However, recent studies suggest a more complex scenario. In Peru, a lower proportion of epiphytes are endemic than confamilial terrestrial species in Bromeliaceae, Piperaceae and Araceae, whereas endemicity is higher in epiphytic orchids than in terrestrial orchids^18^. Family-level patterns seem consistent across comparative studies of range size. For example, epiphytic aroids of the genus *Anthurium* have mean range size eight times larger than terrestrial species^19^, whereas epiphytic orchids of the genus *Galeandra* tend towards smaller ranges than terrestrial congeners^20^. Terrestrial and lithophytic bromeliads are consistently found to have smaller range sizes than epiphytic bromeliads^21,22^. However, angiosperm-wide analyses in the Atlantic Forest of Brazil showed that epiphytes have some of the smallest average ranges among lifeforms^2,22^, contrary to Schimper’s generalization.

Adaptations for tree-to-tree dispersal predispose epiphytes to high dispersal capabilities^21^, contributing to large range sizes^23^. Tree height favours dispersal of wind- or animal-dispersed seeds of canopy-dwelling epiphytes^19,24^. Such adaptations are prevalent in species-rich epiphytic groups, for instance, dust-like seeds in Orchidaceae, plumed seeds in tillandsioid Bromeliaceae and fleshy bird-dispersed fruit in Araceae^7^. Conversely, plant clades resulting from rapid diversification tend to have small-ranged species, especially when speciation arises from isolated small populations^25,26^. Rapid diversification of epiphytic clades may therefore lead to small ranges^27^. These contrasting drivers of the ranges of epiphytes illustrate the need for more nuanced analyses.

Previous studies on the range size of epiphytes focused on particular biogeographical contexts where regionally specific extrinsic factors could differentially impact terrestrial and epiphytic plants, for example historical climatic changes^28^. Often, studies on range size did not capture substantial portions of the taxonomic diversity of epiphytes or account for range size heritability^29^, potentially masking relationships between epiphytism and range size by failing to control for the non-independence of species range sizes caused by shared evolutionary history^30^.

Here, we integrate several data sources to investigate range size and rarity of epiphytic flowering plants at globally and taxonomically inclusive scales. We test if epiphytes differ in geographic range size from terrestrial plants, across angiosperms and in epiphyte-dominated lineages. Applying phylogenetic comparative methods, we test whether epiphytism per se explains observed range differences. Finally, to explore the conservation implications of our results, we estimate the proportion of epiphytes considered rare owing to their small extents of occurrence (EOO), a measure of rarity owing to restricted distribution, or to their low specimen count, which correlates with rarity related to both range size and abundance.

## Results

Two-thirds (65%) of epiphyte angiosperms were native to a single botanical country, the standard unit used to record plant distributions in the World Checklist of Vascular Plants (WCVP)^31,32^. By contrast, 56% of terrestrial angiosperms were native to a single botanical country. Angiosperm epiphytes were absent from 72 botanical countries (mostly in high latitudes or deserts).

**Fig. 1:**
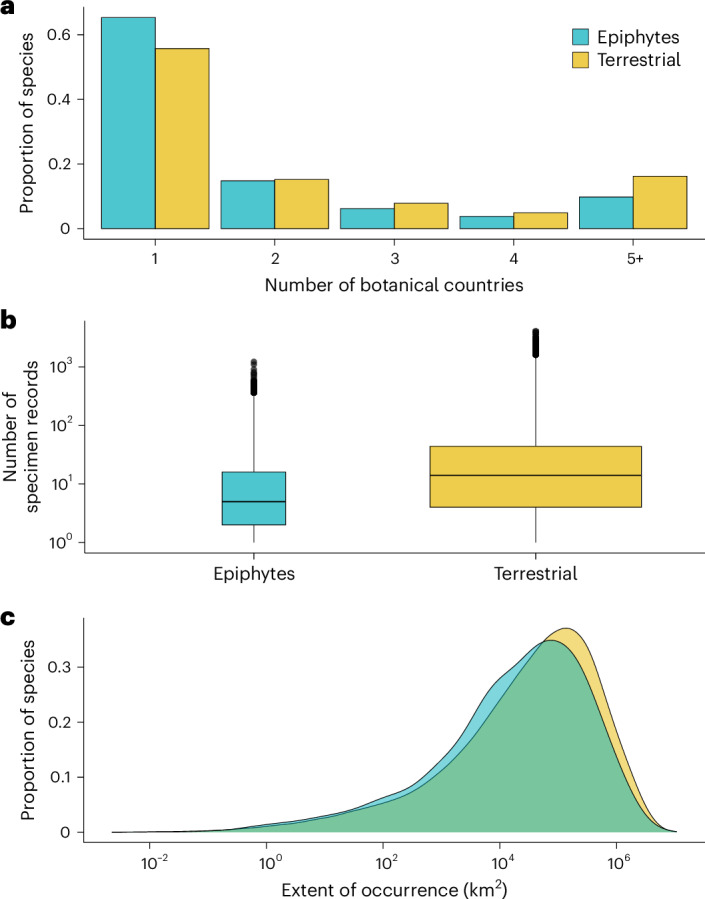
The distribution of three range size metrics in epiphytes and terrestrial plants. Epiphytes tend to occur in fewer botanical countries, have fewer specimen records and have slightly different frequency distributions of EOO compared to terrestrial plants. **a**, Proportion of epiphytes and terrestrial plants native to one to five or more botanical countries. **b**, Minimum, first quartile, median, third quartile, maximum and outliers for the number of specimens transformed at the log_10_ scale, with boxes proportional to the number of species in each group (epiphytes *n* = 20,322 and terrestrial *n* = 224,131). **c**, Frequency distributions of the EOO for epiphytes and terrestrial plants transformed at the log_10_ scale.

As most variation in range size not captured by number of botanical countries is among small-ranged species, particularly those occurring in four or fewer botanical countries, we calculated two additional metrics for angiosperms native to four or fewer botanical countries: sum of unique herbarium specimen records for a species (hereafter, ‘specimen count’) and EOO. After data cleaning and name matching with the phylogenetic trees^33^, our dataset contained specimen counts of 74.1% and EOO of 44.2% of angiosperms. Specimen counts for epiphytes native to four or fewer botanical countries ranged from 1 to 1,224 with a median of five records after cleaning (Fig. 1). For terrestrial plants, specimen counts ranged from one to 4,048 with a median of 14 records. The EOO of epiphytes native to four or fewer botanical countries ranged from 0.01 to 7,431,335 km^2^ (median 29,003 km^2^). For terrestrial plants, EOO ranged from 0.002 to 10,696,300 km^2^ (median 45,880 km^2^).

### Range size and rarity of epiphytes

**Fig. 2:**
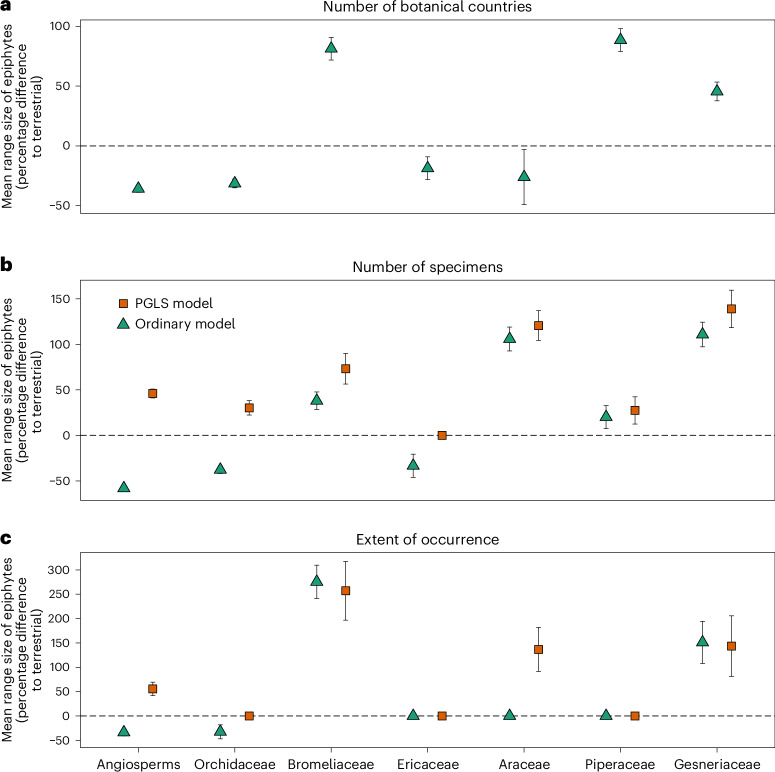
Percentage difference of mean epiphyte range size compared to terrestrial species across three metrics, for ordinary and phylogenetically controlled regression models, for angiosperms and six epiphyte-rich families. Across angiosperms, epiphytes, on average, occur in fewer botanical countries, have fewer specimen records and smaller EOO than terrestrial plants (shown using ordinary, non-phylogenetic regression models), confirming their rarity relative to angiosperms on average. However, counterintuitively, data suggest that the epiphytic lifeform contributes to increasing species ranges, as shown when variation in range size due to phylogenetic relationships is partitioned out to isolate the independent effect of lifeform on range size, using PGLS models. These counterintuitive findings support the epiphytic lifeform as an indicator of rarity as well as the hypothesis that the epiphytic habit promotes dispersal (contributing to large ranges). Within-family patterns of range size variation differ between the six most epiphyte-rich families. **a**–**c**, Mean range size is given as percentage (%) difference to terrestrial species with 95% CI for the three metrics: number of botanical countries (PGLS not performed, *n* = 330,987) (**a**); specimen count (*n* = 244,453) (**b**); and EOO (*n* = 145,898) (**c**). The horizontal dotted line represents the baseline of no significant difference between epiphytes and terrestrial plants. See Supplementary Table 6 for sample size of epiphyte-rich families for each metric.

**Extended Data Fig. 1:**
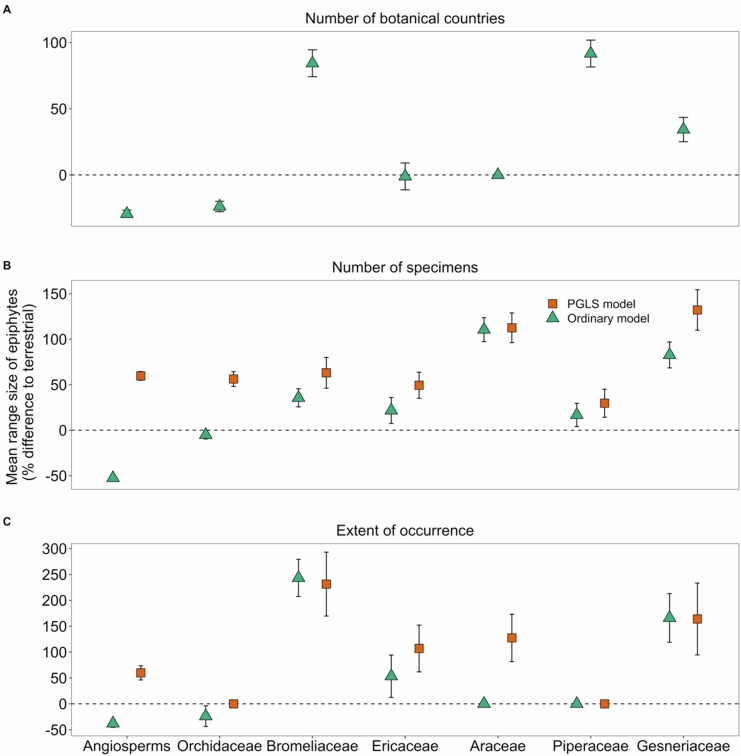
Sensitivity analysis restricted to species which have the mean latitude of their distribution in the tropics. Summary of results for sensitivity analysis restricted to species which have the mean latitude of their distribution, measured as botanical countries, in the tropics (between latitudes 23.3 N and 23.3 S, n = 195,273). Mean range size is given as percentage (%) difference to terrestrial species with 95% confidence intervals for the three metrics **a**) number of botanical countries (PGLS not performed); **b**) specimen count; **c**) extent of occurrence. The horizontal dotted line represents the baseline of no significant difference in the percentage difference in range size between epiphytes and terrestrial plants.

**Extended Data Fig. 2:**
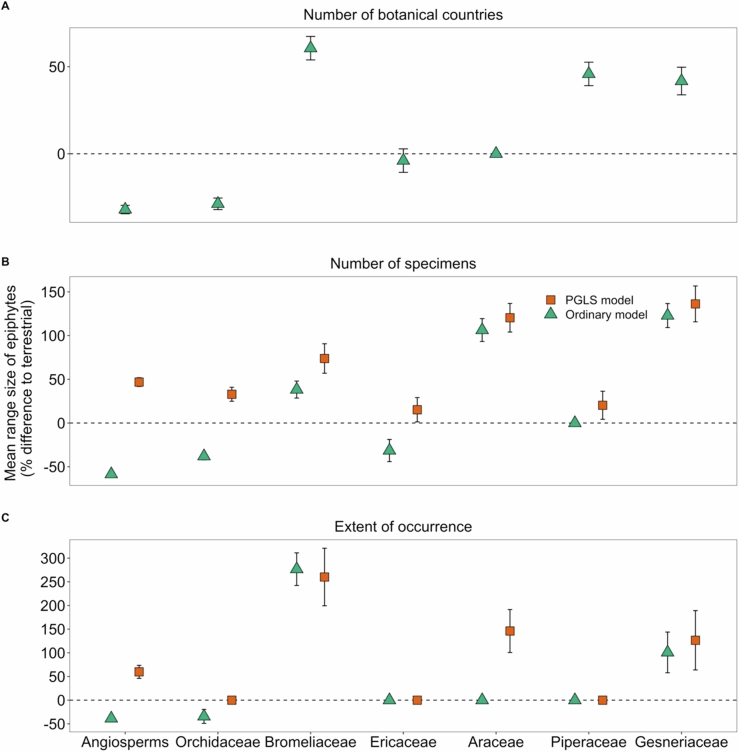
Sensitivity analysis restricted to species which do not occur on oceanic islands. Summary of sensitivity analysis restricted to species which do not occur on any of the 55 botanical countries which are composed of one or more oceanic islands (n = 308,105). Mean range size is given as percentage (%) difference to terrestrial species with 95% confidence intervals for the three metrics **a**) number of botanical countries (PGLS not performed); **b**) specimen count; **c**) extent of occurrence. The horizontal dotted line represents the baseline of no significant difference in the percentage difference in range size between epiphytes and terrestrial plants.

Across angiosperms, our regression models showed that epiphytes had significantly smaller average ranges across all three metrics when modelling range size as a function of lifeform (terrestrial or epiphytic) and not controlling for the effect of shared ancestry (Fig. 2). Epiphytic species are native to, on average, 36% (95% confidence interval (CI) 34–38%) fewer botanical countries, have 58% (95% CI 57–59%) fewer specimens and have 34% (95% CI 31–36%) smaller EOO than terrestrial species. The similarity in effect sizes supports using each metric as a measure or proxy of range size. When restricting analyses to tropical species, effect sizes varied by <10 percentage points for all three metrics, demonstrating that global patterns across angiosperms are not confounded by the primarily tropical distribution of epiphytes (Extended Data Fig. 1). Excluding angiosperms occurring on oceanic islands had a similarly small effect: <6 percentage points across metrics (Extended Data Fig. 2).

Twenty-four angiosperm families include ten or more epiphytic species (Supplementary Table 1). Range size of lifeforms within these families showed considerable variation from the angiosperm-wide trend and between families (Supplementary Tables 2–4). Epiphytes had larger mean specimen count and EOO than confamilial terrestrial species in more cases than the opposite.

**Fig. 3:**
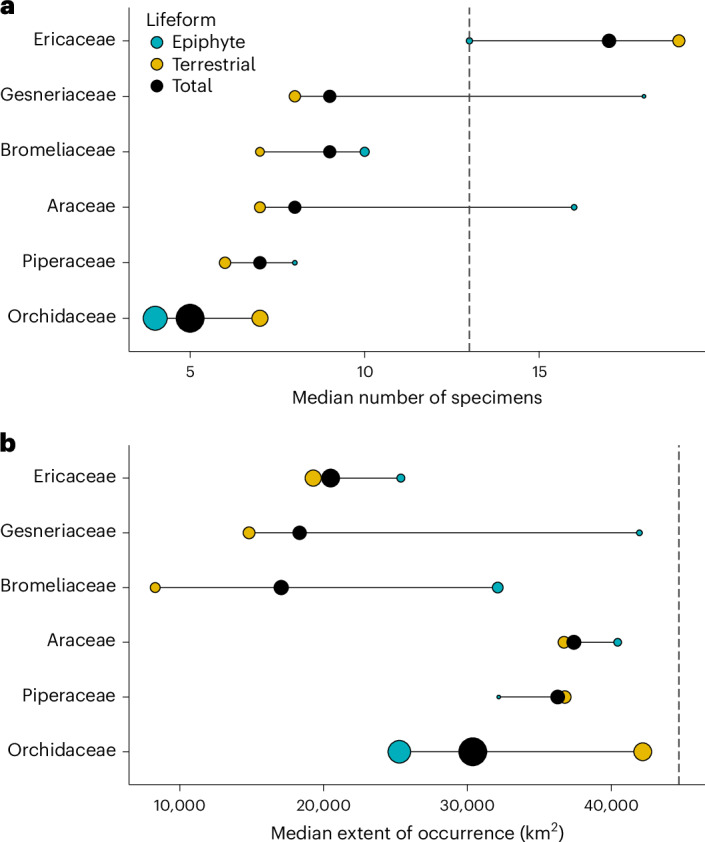
Median range size of epiphytes, terrestrial species, and all species in the six most epiphyte-rich angiosperm families. When considering species native to four or fewer botanical countries, the median range size of epiphyte-rich families is typically smaller than the angiosperm-wide median range size. **a**,**b**, Bubbles show median range size measured as specimen count (**a**) and EOO of epiphytic, terrestrial and all species (**b**) for each of the six most epiphyte-rich families. The median range size of angiosperms native to four or fewer botanical countries (EOO = 44,705 km^2^, specimen count = 13) is the vertical dotted line. Bubble size is proportional to the number of species in each group.

Although 56 angiosperm families contain epiphytic taxa, 94% of epiphyte species are concentrated in six families: Orchidaceae (*n* = 20,698 epiphyte species), Bromeliaceae (*n* = 1,918), Ericaceae (*n* = 872), Araceae (*n* = 766), Piperaceae (*n* = 699) and Gesneriaceae (*n* = 644). Comparison of range size between lifeforms within these families showed that the only family in which epiphytes had fewer specimens and smaller EOO than terrestrial species was the species-rich, mostly epiphytic Orchidaceae (Fig. 2, ordinary model), which contains 76% of angiosperm epiphyte species. In Bromeliaceae and Gesneriaceae, epiphytes had more specimens and larger EOO than terrestrial confamilial species. However, these differences are largely driven by the small range sizes of terrestrial family members, particularly the extremely small ranges of terrestrial bromeliads (Fig. 3). In the remaining epiphyte-rich families, mean specimen count was either larger (Araceae and Piperaceae) or smaller (Ericaceae) for epiphytes, whereas mean EOO did not differ between lifeforms (Fig. 2). Sensitivity analyses restricted to tropical species yielded similar results, with appreciable differences in effect size only in Orchidaceae and Ericaceae (Extended Data Fig. 1). Epiphytic orchids had slightly smaller mean EOO (no difference in specimen count) than terrestrial species, whereas epiphytic Ericaceae exhibited larger EOO and specimen count than terrestrial species.

Species in epiphyte-rich families (including both epiphytic and terrestrial species) have, on average, 53% (95% CI 52–54%) fewer specimens and 42% (95% CI 40–44%) smaller EOO than species in other angiosperm families. Five of the six most epiphyte-rich families had median specimen counts smaller than the angiosperm-wide average (except Ericaceae, Fig. 3). All epiphyte-rich families had smaller median EOO than the angiosperm-wide median. These results provide evidence that belonging to an epiphyte-rich family has similar or stronger effects on range size than does being epiphytic.

Of 8,170 epiphyte species for which EOO was estimated, 45% had EOO <20,000 km^2^, the threshold for categorization as ‘vulnerable’ following Red List criterion B1 (ref. ^5^). These species could potentially qualify for a threatened category under criterion B but would need to meet additional subcriteria to be categorized as threatened in a Red List assessment^5^. Of 20,322 epiphyte species for which specimen count was estimated, 51% had five or fewer specimens. By contrast, 39% of the 137,728 terrestrial species with EOO estimates had EOO <20,000 km^2^ and 29% of the 224,131 terrestrial species studied had five or fewer specimens, showing that epiphytes had a consistently higher proportion of rare species compared to terrestrial plants. The global proportion of species which meet these thresholds for rarity^5,34^ is still unknown because 27% of angiosperms were not included in this analysis, as a result of their lack of digitally available specimen records (12%) or their occurrence in five or more botanical countries (16%).

### Effect of epiphytism on range size

Controlling for covariation due to shared ancestry revealed that the tendency for angiosperm epiphytes to have small ranges is not caused by epiphytism. The phylogenetically independent effects associated with the epiphytic habit contributed to larger range sizes: on average, 46% (95% CI 43–49%) more specimens and 54% (95% CI 47–61%) larger EOO (Fig. 2) than terrestrials. Uncertainty associated with the imputation approach to add missing species to the phylogenetic trees was consistently low (Supplementary Tables 2 and 3).

The phylogenetically independent effects of epiphytism were associated with larger mean EOO and mean specimen counts in three epiphyte-rich families: Bromeliaceae, Araceae and Gesneriaceae (Fig. 2). Other families showed no effects of epiphytism on mean EOO, but either positive effects (Orchidaceae and Piperaceae) or no effect (Ericaceae) on specimen counts. However, in tropical-only analyses, epiphytic Ericaceae showed larger EOO and specimen count, exhibiting a similar pattern to the primarily tropical families Bromeliaceae and Gesneriaceae (Extended Data Fig. 1). In phylogenetic models, epiphytism was not associated with decreases in mean EOO or specimen count for 23 of the 24 families with ten or more epiphytic species (exception being the small Neotropical family Schlegeliaceae), indicating that epiphytism is rarely functionally associated with range sizes smaller than those of confamilial terrestrial species (Supplementary Tables 2 and 3).

## Discussion

Analyses controlling for phylogenetic relatedness reveal that epiphytes are associated with larger range sizes than closely related terrestrial plant species. By conducting phylogenetically controlled analyses, we infer the effect of evolution on the relationship between epiphytism and range size. The small ranges of epiphytes result from their concentration in a few species-rich lineages, where diversification and trait evolution have promoted small ranges. Epiphytism itself does not cause small ranges.

Traits predisposing species to small range sizes may be prevalent in epiphyte-rich clades. The epiphytic habitat is characterized by several key challenges, including limited and variable water supplies^35^, low substrate stability^36^ and discontinuous habitat^37^. Epiphytes possess unique trait syndromes compared to ground-rooted plants^38^ and diverse morphological and physiological traits observed in epiphytes are interpreted as adaptations for arboreal life^7^. However, traits advantageous in the epiphytic habitat are often shared with closely related terrestrial species and thus may not necessarily be adaptations for epiphytism. For instance, structures associated with desiccation avoidance or tolerance in epiphytic taxa often occur across entire clades containing epiphytes and non-epiphytes. The absorptive velamen radicum root epidermis, typically considered an adaptation in epiphytic orchids and some aroids, is found throughout monocotyledons, including >160 terrestrial orchid genera^39^. The water-impounding tank structures (phytotelmata) and absorptive leaf scales of bromeliads characterize both terrestrial and epiphytic taxa within specific subclades^40^. The abundance of taxa exhibiting facultative epiphytism^8^ and numerous apparent gains and losses of epiphytism in epiphyte-rich clades such as orchids also indicate that epiphytism may be an evolutionarily labile trait within certain lineages^10,41^.

Epiphytic and terrestrial taxa within certain clades are not only morphologically similar, but also ecologically similar. Lithophytes, plants which grow on rocks (here included in terrestrial plants), face challenges associated with growth on largely impenetrable substrates, such as those faced by epiphytes. The lithophytic lifeform is common in epiphyte-rich families, including Orchidaceae (for example, *Epidendrum*, *Laelia* and *Cyrtopodium*), Bromeliaceae (for example, *Encholirium*, *Pitcairnia* and *Tillandsia*), Araceae (for example, *Anthurium*) and Gesneriaceae (for example, *Sinningia*)^21,42^. Many species in these families grow as either epiphytes or lithophytes^7^, exemplifying the similarity of these habitats and their demands. Thus, epiphyte-rich families may possess traits adapted for stressful conditions including, but not limited to, the epiphytic habitat. The tendency for entire epiphyte-rich families to have small ranges may be linked to their specialism in stressful habitats. Niche breadth is positively correlated with range size^43^ and other groups of substrate-specialist plant species such as those restricted to rocky outcrops^2^ and serpentine soils^44^ also tend towards small range sizes.

Although epiphytism itself may not be functionally related to small ranges, evolution of key traits underpinning colonization of and radiation within the epiphytic habitat may indirectly yield the small ranges observed in epiphyte-rich clades by triggering speciation and increasing net diversification. Epiphyte-rich clades are notably speciose at several taxonomic scales: Orchidaceae (70% epiphytic) is the most speciose plant family and Bromeliaceae (54% epiphytic) is among the most species-rich in the Neotropics. Epiphyte-dominated genera are among the most species-rich in plants, for example *Bulbophyllum* (Orchidaceae), 2,106 species; *Peperomia* (Piperaceae), 1,409 species; and *Anthurium* (Araceae), 1,137 species^32^. Studies integrating phylogenetic and trait data link the remarkable species diversity of orchids and bromeliads to accelerations in diversification exceeding average rates for angiosperms. In Bromeliaceae, evolution of phytotelmata, wind- and bird-dispersed seeds and epiphytism are linked to accelerated diversification^45^. In orchids, diversification is linked to the evolution of pollinia, Crassulacean acid metabolism photosynthesis and epiphytism^46^. Both families diversified in tropical cordilleras characterized by topographical complexity and barriers to gene flow, perhaps fostering genetic isolation of small populations, begetting many closely related, small-ranged species^45,46^.

The contribution of epiphytism to large range sizes may be linked to the dispersal abilities of epiphytes. Dispersal ability is positively associated with range size in vascular plants, albeit variably between clades^23^. Epiphyte-rich lineages are characterized by seed types adapted for tree-to-tree dispersal, including light, wind-dispersed seeds (for example, dust-like seeds in orchids, plumed seeds in tillandsioid bromeliads and gesneriads) and fleshy, bird- or bat-dispersed seeds (for example, Bromelioideae bromeliads and *Anthurium*, Araceae)^7,10,17,47,48^. Seed weight in epiphytes is bimodally distributed, consistent with hypotheses that light wind-dispersed and heavier, fleshy bird-dispersed seeds are effective for tree-to-tree dispersal. Yet epiphytes do not generally have smaller seeds than confamilial terrestrial plants, evidence that closely related epiphytic and terrestrial taxa exhibit similar traits^48^. Nonetheless, dispersal of anemochorous seeds is probably more successful from the canopy than from the ground, thereby predisposing epiphytic taxa to larger ranges than morphologically similar, closely related terrestrial taxa^19^. Bird-dispersed epiphyte seeds probably reach greater distances assuming canopy-inhabiting birds and bats forage over greater distances than forest-floor birds^18,45^.

In certain families, the much larger ranges of epiphytes compared to terrestrial species could be partly attributable to the exceptionally small ranges of their terrestrial species. Notably, terrestrial bromeliads and gesneriads have median EOO five and three times smaller than the angiosperm-wide median, respectively (Fig. 3). Terrestrial species endemism is frequently driven by substrate specialization^49^, whereas evidence is lacking for host tree specificity in vascular epiphytes^50^. Substrate-driven endemism may be particularly marked in lithophytes on highly fragmented and isolated island-like rock outcrops such as inselbergs^42^. In Brazil and Madagascar, centres of epiphyte diversity^9^, inselberg floras exhibit exceptional endemism richness^42^. Thus, the small ranges of some terrestrial species in epiphyte-rich families, including bromeliads and gesneriads, may be driven by lithophytes and other endemics of isolated and fragmented habitats.

Angiosperm epiphytes are largely restricted to tropical regions, which are experiencing some of the highest rates of habitat loss and degradation globally^51^. Epiphytes may be particularly vulnerable to extinction^52^, even when controlling for range size^2^. They are most diverse in montane cloud forests^10^, ecosystems considered particularly vulnerable to climate change effects due to altitudinal shifts and reductions in cloud formation^15^. Moreover, their dependence on atmospheric moisture and microclimate specialization makes epiphytes highly sensitive to even small changes in humidity and therefore likely to be among the organisms most negatively affected by climate change in montane forests^15^. Epiphytes are susceptible to disturbance and changes to the composition of host trees. For instance, hurricane damage can have direct (for example, branch fall) and indirect effects (for example, altered microclimates) on epiphyte communities^53^ potentially leading to long-term population declines^54^. Selective logging of large, old trees may remove hosts bearing particularly diverse epiphyte assemblages^55^. However, in the Atlantic rainforest of Brazil, lifeform predicts extinction risk only in non-phylogenetic models^2^, suggesting that vulnerability may be a characteristic of entire epiphyte-rich lineages^22^, consistent with the relationship between epiphytism and range size demonstrated here. Many epiphytes and their close terrestrial relatives exhibit slow life histories, predisposing them to slow population recovery following disturbances^56–58^. Species in epiphyte-rich clades are also particularly threatened by overcollection for horticultural use^59^, a threat potentially exacerbated by slow population growth^58^.

Our findings that 45% of epiphytes have EOO <20,000 km^2^ and 51% have five or fewer specimens are congruent with automated assessments of extinction risk which predict similarly high levels of extinction risk and rarity in epiphytes^60^. This is cause for grave concern for the persistence of these species. Despite their unusual ecology, inherently small ranges and apparent heightened vulnerability to extinction, the conservation status of most epiphytes has not been evaluated: only 7% of epiphytic angiosperms have Red List assessments, fewer than half the proportion of angiosperms that have been assessed^6^. Some lineages identified here as having particularly small ranges, such as bromeliads and epiphytic orchids, are particularly poorly represented on the Red List^6^. Red Listing of epiphytes and epiphyte-rich lineages should be prioritized to enhance our understanding of the threats they face and inform conservation action for the high proportion probably threatened with extinction.

## Methods

### Data

The WCVP is the first comprehensive, continually updated source for vascular plant species names and geographic distributions^32^. We used WCVP (special issue version^61^) to obtain taxonomic and geographic distribution data for 330,087 accepted angiosperm species not of hybrid origin and with known distributions. We intersected WCVP and EpiList 1.0, a comprehensive list of epiphytes, to categorize 27,184 angiosperm species as epiphytes for this analysis^8^. We included all the species listed on the WCVP as epiphytic (using the mapping from ref. ^62^) as well as any species on the EpiList that were not already labelled as an epiphyte in the WCVP^8,32,62^. Species listed as hemi-epiphytes, which germinate epiphytically but later root in the ground, were excluded from this analysis because their ecophysiology differs from true epiphytes^63^. Restricting our analysis to angiosperms prevented overcomplicating inferences concerning determinants of range size by excluding pteridophytes, which have diverging physiology and distribution patterns^7,9^.

Occurrence records were obtained from the Global Biodiversity Information Facility (GBIF.org; see Supplementary Table 5 for download citations) using the R package rgbif^64^. Only herbarium specimen-based occurrence records were used for our calculations because they represent verifiable evidence of species occurrences and underpin accurate estimations of species distributions even at relatively low sampling densities^65,66^.

### Data cleaning

**Extended Data Fig. 3:**
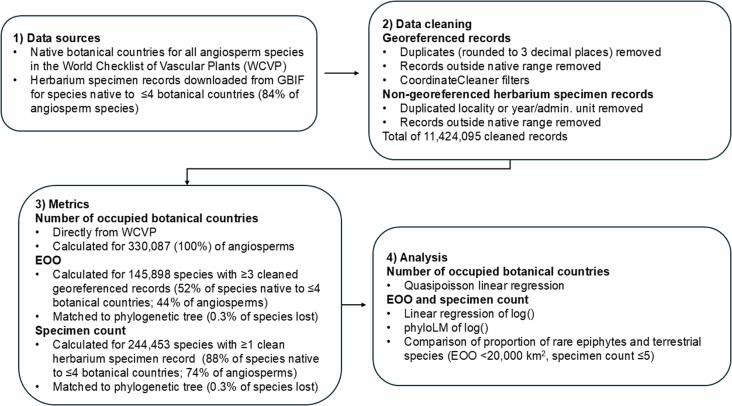
Workflow for development of datasets. Workflow depicting the different development stages of the datasets, data cleaning and primary analyses: 1) the data sources used, 2) data cleaning measures, 3) calculation of range size metrics, and 4) analyses.

Spatial and taxonomic biases, coordinate inaccuracies and duplication of records are well-documented issues with aggregated biodiversity databases, including GBIF^67,68^. To mitigate these issues, we used distinct data-cleaning procedures for georeferenced and non-georeferenced records (Extended Data Fig. 3). Among georeferenced occurrence records, we detected records of the same species with identical coordinates (rounded to three decimal places), removed duplicates and retained at most one record per 110 m^2^ for each species. We used the R package rWCVP to remove records outside the reported native range at botanical country level for each species^69^. We removed records with low precision (coordinate uncertainty >100 km) and applied automatic filters from the R package CoordinateCleaner to remove potentially erroneous or unfit records^70^. We discarded records where latitude equals longitude (or any equals 0), or coordinates corresponded to country centroids, capitals or biodiversity institutions. We removed records that were erroneously downloaded as a result of fuzzy name matching (for example, genus-level records).

**Extended Data Fig. 4:**
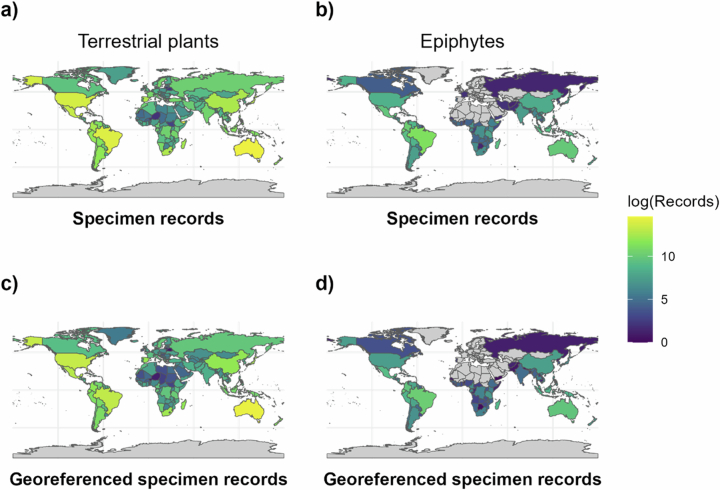
Global maps of the logarithm of the number of records. Global maps of the logarithm of the number of records in our cleaned dataset per country for **a**) specimen records of terrestrial plants, **b**) specimen records of epiphytes, **c**) georeferenced specimen records of terrestrial plants, and **d**) georeferenced specimen records of epiphytes. Specimen counts were calculated using all specimen records whereas EOOs were calculated using georeferenced specimen records only.

To clean non-georeferenced occurrence records, we removed records of species with duplicated collection locality or duplicated year and administrative unit (stateProvince in GBIF) as these probably represent duplicates of the same population or collection event, respectively. Non-georeferenced records outside native countries of occurrence of species were removed by mapping botanical countries to ISO country codes in GBIF records and removing records from outside the native ranges of species as recorded in WCVP. In total, 11,424,095 occurrence records (34% of the total number downloaded from GBIF) were retained after data cleaning, of which 5,882,511 were georeferenced and 5,541,584 were non-georeferenced (Extended Data Fig. 4).

### Range sizes and phylogeny

We obtained the number of native, occupied botanical countries for 330,087 accepted angiosperm species directly from WCVP. Botanical countries is the standard unit for recording plant distributions in WCVP following the World Geographical Scheme for Recording Plant Distributions^31^. Globally, the 368 botanical countries mostly correspond to political countries, although some large countries are split into smaller units, and geographically disjunct areas (for example, islands) may be recognized as distinct botanical countries, reflecting their phytogeographical differences^31^. The number of botanical countries captures variation in EOO well among large-ranged species and probably around half the total variation in EOO^52^. However, the coarse resolution of botanical countries misses range size variation among small-ranged species. To account for this variation, we calculate two additional metrics of range size for species occurring in four or fewer botanical countries (84% of all angiosperm species): sum of all unique herbarium specimen records for a species (specimen count) and EOO calculated from georeferenced herbarium specimen records only. Most occurrence records are of common species^66^ because most plant species are rare^34^. Excluding the most widespread angiosperms from occurrence record-based analyses (that is, metrics EOO and specimen count) therefore substantially reduced the computational load associated with downloading and cleaning occurrence data while maximizing the number of species included. Because up to four botanical countries can meet at a single point, species with extremely small EOO can be recorded in up to four botanical countries. Thus, our threshold of including species occurring in up to four botanical countries should capture nearly all species with very small ranges.

The EOO was estimated using cleaned georeferenced occurrence records for species having three or more georeferenced records. Using the R package rCAT, we calculated EOO as the minimum convex polygon encompassing all occurrence points for a species^71^. We used specimen count as a complementary metric of range size and a correlate of the abundance of each species. We used cleaned georeferenced and non-georeferenced occurrence records to calculate specimen counts. Omitting non-georeferenced records would risk overestimating the rarity of many species^66^, as 49% of the cleaned GBIF records are not georeferenced. Using specimen count also helped to maximize the number of species included in our study and minimizes bias against species lacking georeferenced records, which are generally poorly known and rare: 58,974 angiosperm species occurring in four or fewer botanical countries had only non-georeferenced GBIF records. This is particularly the case for epiphytes; a disproportionate number lack the three georeferenced records required for EOO estimation. As a result, epiphytes are better represented in our specimen count dataset (8.3% of species) than in the EOO dataset (5.6% of species, Supplementary Table 6). Studies using number of specimens as a correlate of global abundance of individual species^34^ and range size^65^ report good performance against expert-backed assessments.

To control for phylogenetic distances between species spanning the angiosperm clade, we used a set of 100 species-level phylogenetic trees for angiosperms prepared with 329,798 accepted species^33^. This tree uses the GBMB phylogenetic tree of ref. ^72^ as a backbone, updated to reflect recent taxonomic changes to angiosperm families, genera and species as published in WCVP (v.6). Missing genera and species were randomly added at the appropriate crown nodes, producing 100 trees by repeating the imputation stage 100 times to account for uncertainty in phylogenetic placement of these taxa. All names in the phylogenetic trees are standardized to WCVP, facilitating name matching between range size data and tree tip labels for phylogenetic regression. The difference in species numbers between the phylogenetic trees and the distribution data is due to nomenclatural and taxonomic changes in the WCVP which took place between the version of the WCVP used to build the trees (v.6) and the special issue version^61^ used for this analysis.

After data cleaning and name matching with a phylogenetic tree, we achieved 100% coverage of 330,087 angiosperm species and 27,184 epiphytes with one range size metric (number of botanical countries; used only for ordinary regression), 74.1% coverage (244,453 species) of angiosperms and 74.8% (20,322 species) of epiphytes with two range size metrics (botanical countries and specimen count) and 44.2% (145,898 species) coverage of angiosperms and 31.20% (8,170 species) of epiphytes with all three range size metrics. Our omission of species occurring in five or more botanical countries from specimen count and EOO metrics accounts for 16% of coverage loss for these metrics. Only 0.3% of species with specimen count or EOO data could not be matched to our phylogenetic trees and were therefore omitted from these datasets. The remainder of missing species in the specimen count analysis did not have any herbarium specimen records in GBIF after data cleaning, while the remainder of species missing in the EOO analysis did not have enough cleaned georeferenced GBIF records to calculate a minimum convex polygon.

The percentage of epiphytes for which EOO could be calculated varied across the six most epiphyte-rich families, being the lowest in Orchidaceae (26%) and highest in Araceae (72%). Most of this variation was due to species, especially orchids, having fewer than three georeferenced records, preventing the calculation of EOO. The percentage of epiphytes for which specimen counts were available was more consistent across families (Supplementary Table 6). Across epiphyte-rich families, coverage by at least one GBIF-derived metric was lowest for Orchidaceae (80%) and highest for Bromeliaceae (94%).

### Data quality and caveats

Two of the three metrics we used as surrogates for range size rely directly on data from GBIF, widely recognized as the most comprehensive species occurrence resource^73^ but also as containing many errors and biases^67,74^. Among the many documented limitations of GBIF data for plants, taxonomic inaccuracies and uneven geographic coverage are arguably most likely to affect our study. We sought to minimize the former by using only herbarium specimen-based records, which are auditable and more likely to bear authoritative identifications^65^. This approach may have resulted in underestimation of range size for some species. However, we refrained from addressing potential geographic gaps by adding epiphyte data from the published literature or herbaria as this would risk distorting the comparison between epiphyte and non-epiphyte specimen numbers. The broad congruence between our three range size metrics, one of which (botanical countries) is not derived from GBIF data, gave us confidence that, although far from perfect, they are adequate to support an initial overview at the global level, which more detailed studies at regional levels should test.

### Statistical analyses

Regression methods were used to test the relationships between lifeforms (epiphyte versus terrestrial) and each of the three range size metrics across angiosperms. We also investigated differences in range size and the effect of epiphytism within each of the 24 families with ten or more epiphytic species (Supplementary Table 1) using each of the three metrics (Supplementary Tables 2–4). All statistical analyses and data manipulation were conducted in the R environment^75^ (v.4.1.2). We conducted a sensitivity analysis restricted to angiosperms for which the mean latitude of their distribution, measured as botanical countries, is in the tropics (*n* = 195,273; Extended Data Fig. 1) to test whether our results are confounded by the largely tropical distribution of epiphytes^9^ and/or the tendency for range size to increase with latitude^76^. A further sensitivity analysis excluded 21,982 angiosperm species occurring on oceanic islands (*n* = 308,105; Extended Data Fig. 2), which have different biogeographical and evolutionary histories from those of continental islands and mainland regions^77^.

All three range size metrics were highly positively skewed and therefore non-normal, owing to the large proportion of species with very small ranges. For both specimen count and EOO, we performed ordinary least squares (OLS) regression using log-transformed variables to meet the normality assumption. OLS regression does not account for phylogenetic signal, so it allows the comparison of unweighted range size values. For example, this analysis can provide evidence that epiphytes have smaller ranges than the average terrestrial, offering useful context for comparison and information for species-level conservation prioritization (where species are typically treated as independent units). For specimen count and EOO metrics, we performed phylogenetic generalized least squares (PGLS) regression in addition to OLS regression. Using phylogenetic regression, we estimated the independent effect of lifeform (epiphyte versus terrestrial) on range size by controlling for the non-independence between species caused by shared ancestry. Since closely related species are more likely to have similar range sizes than expected by chance^78^, phylogenetic regression is appropriate for testing the causality of epiphytism on range size^79^. Owing to the low variation and prevalence of species native to a single botanical country (56%) we could not satisfactorily transform the data for botanical countries to meet the assumptions of a linear model. We therefore used a generalized linear regression for this metric, with a quasi-Poisson model to account for overdispersion in the relationship between number of occupied botanical countries and lifeform. We did not perform the phylogenetic regression for the botanical countries because implementing the PGLS method we used was unsuitable for a quasi-Poisson model.

We used the function phylolm from the R package phylolm to carry out the phylogenetic regressions^80^. Phylolm uses the PGLS method to correct the error structure of the residuals of the linear model to account for covariation due to shared ancestry. We used Pagel’s *λ* to estimate the strength of the phylogenetic signal in the residuals of the model. Pagel’s *λ* is derived through maximum likelihood estimation and is multiplied by the expected variance–covariance matrix to account for the strength of the phylogenetic signal in the data, assuming Brownian motion as the mode of evolution^81^. Values of *λ* approaching 1 indicate very strong phylogenetic correlation between species while values of *λ* close to 0 indicate weak phylogenetic correlation. The phylolm function calculates *λ* on the basis of the residual errors of the model and does not reflect the phylogenetic signal of the response variable itself, but rather of the relationship between the predictor(s) and response variable. Range size heritability varies between groups^82^ and phylogenetic signal in the relationship between lifeform and range size is unlikely to be fixed across angiosperms: Pagel’s *λ* accounts for this variability. Phylogenetic regression models were run for the entire angiosperm dataset using each of the 100 phylogenetic trees^33^. The mean and standard error of parameter estimates were calculated to account for uncertainty in the phylogenetic placement of imputed taxa^83^.

To investigate how the range size of epiphyte-rich groups compares to other angiosperm lineages, we tested if the mean specimen count and EOO for species in each of the six most epiphyte-rich families differed from that of species in other families. The most epiphyte-rich families were Orchidaceae (*n* = 20,698 epiphyte species), Bromeliaceae (*n* = 1,918), Ericaceae (*n* = 872), Araceae (*n* = 766), Piperaceae (*n* = 699) and Gesneriaceae (*n* = 644). These families include >90% of epiphyte species and represent lineages that have effectively radiated in the epiphyte niche^10^. Focusing on the six most epiphyte-rich families maximizes the number of epiphytic species with available range size data for within-family analyses, allowing us to examine characteristics of clades which might explain the patterns we uncovered. For each family, we plotted the percentage difference between mean epiphyte range size and terrestrial range size across all three metrics for both phylogenetic and ordinary regression models, to facilitate cross-metric comparison of the effect of epiphytism on range size in these lineages (Fig. 2).

### Rarity

Rarity is a multidimensional concept^3,4^ and, although a universal definition is lacking, rare species are typically considered as those having restricted distributions, low abundance or both^3^. The three metrics of range size we calculate here (number of native botanical countries, EOO and specimen count) can be used to estimate which species are rare as a result of having restricted distributions, a major axis of rarity^3,4^ (Table 1). EOO, in particular, is widely used as a conservation-relevant metric of range size, as it reflects the spread of risk across a species range^84^. Specimen count, in addition to its use as a correlate of range size^65^, also serves as a proxy for the relative abundance of species^34^. All else being equal, the less abundant a species is, the lower the probability that it will be collected, resulting in fewer herbarium specimen records in GBIF. Specimen count therefore captures an additional facet of rarity than our other metrics and can be used to detect species which are rare but that do not necessarily have small EOO. The number of native botanical countries is a comprehensive metric of range size for angiosperms, which allows us to pinpoint potentially small-ranged species. However, it is a coarse measure and is therefore not as informative for testing the proportion of rare species as our other metrics.

**Table 1:** Summary of how each of our metrics measure range size or captures abundance, why we selected each metric and the thresholds we used to compare levels of rarity between epiphytes and terrestrial plants

Metrics	Number of botanical countries	EOO	Specimen count
Used as a range size metric?	Yes^52,85^	Yes^1,5^	Yes^65^
Why we use it as a range size metric	Comprehensive, available for all angiosperms Helps pinpoint potentially small-ranged species (species in up to four botanical countries may have very small ranges)	Frequently used metric for geographic range size, including by IUCN Red List (reflects risk spread) More precise than botanical countries Captures more variation between species in up to four botanical countries	Available for more species than EOO (those with fewer than three georeferenced records) More inclusive of poorly known species which tend to be rare and are often excluded from studies due to lack of a suitable metric
Used in other studies to capture abundance?	No	Not found	Yes, but specimens plus observations^34^
How it captures aspects of rarity other than range size?			Low abundance results in low probability of collection, thus low specimen number
Why we use it to capture aspects of rarity other than range size			Available for many species, including very poorly known ones which tend to be rare, and are often excluded from studies due to lack of a suitable metric
Thresholds for rarity		20,000 km^2^ (ref. ^5^)	Specimen count five or fewer^34^

To explore our findings in a conservation context, we calculated the proportion of epiphyte and terrestrial species which can be considered rare on the basis of their small EOO or specimen count. We consider species to be rare if they have EOO <20,000 km^2^, thereby meeting the threshold for a threatened category under Red List criterion B1 (ref. ^5^), or if they have five or fewer specimens, using the threshold for rarity based on number of records described by ref. ^34^. An important caveat is that, whereas we only use herbarium specimen records, the threshold of ref. ^34^ was derived from a dataset including observation records in addition to specimens. However, given that we are comparing the proportion of species with five or fewer specimens between epiphytes and terrestrial species, our use of their threshold is not invalidated. However, the true number of species with five or fewer specimens would be lower if we included observation records. We calculated the proportion of epiphytes assessed on the IUCN Red List by searching the Red List website^6^ for all plant assessments with plant and fungal growth form = ‘Epiphyte’ in June 2022 and dividing the number of assessed species (1,982) by the total number of epiphytes in our dataset (27,184).

### Reporting summary

Further information on research design is available in the Nature Portfolio Reporting Summary linked to this article.

## Supplementary information

Supplementary Information Supplementary Tables 1–6

Reporting Summary

## Data Availability

The version of the WCVP used in our study is available at 10.34885/rar9-jx25. Lifeform data were obtained from the EpiList 1.0 (10.1002/ecy.3326). Occurrence records were downloaded from GBIF (https://www.gbif.org/; Supplementary Table 5 gives GBIF download DOIs). The data used for the analysis are available via Zenodo at 10.5281/zenodo.15122031 (ref. ^86^).

## Code availability

The R scripts used for the analysis are available via Github at https://github.com/vsvahn/GlobalEpiphytes.
