# Supplementary material for: Geographic range size and rarity of epiphytic flowering plants
Source: Nat Plants. 2025 Jun 13;11(7):1380–9. doi: 10.1038/s41477-025-02022-9 (PMC12283404; doi:10.1038/s41477-025-02022-9)
Supplement: Supplementary file 1 — Supplementary Tables 1–6 [file 41477_2025_2022_MOESM1_ESM.pdf]

nature plants

Supplementary information https://doi.org/10.1038/s41477-025-02022-9

Geographic range size and rarity of epiphytic flowering plants

In the format provided by the authors and unedited

Supplementary information

Table S1: Summary of the number and proportion of epiphytic species in angiosperms and each of the twenty-four angiosperm families with 10 or more epiphytic species.

Group	Number of epiphytes	Epiphyte proportion
Angiosperms	27,184	0.082
Orchidaceae	20,698	0.703
Bromeliaceae	1,918	0.544
Ericaceae	872	0.196
Araceae	766	0.186
Piperaceae	699	0.183
Gesneriaceae	644	0.173
Melastomataceae	362	0.062
Apocynaceae	247	0.038
Rubiaceae	189	0.013
Cactaceae	137	0.079
Cyclanthaceae	60	0.26
Begoniaceae	59	0.031
Araliaceae	54	0.033
Balsaminaceae	47	0.044
Urticaceae	41	0.020
Asteraceae	39	0.001
Solanaceae	35	0.013
Crassulaceae	34	0.021
Campanulaceae	33	0.014
Zingiberaceae	33	0.018
Nepenthaceae	30	0.170
Lentibulariaceae	28	0.067
Asparagaceae	25	0.008
Schlegeliaceae	16	0.432

Table S2: Estimated effects of epiphytism on range size measured as $\log_{10}$(Specimen count) according to ordinary linear regression models and phylogenetic generalised least squares regression models for angiosperms, angiosperms within families containing at least one epiphytic species and each family with more than ten epiphyte species. Coefficients show estimated difference to $\log_{10}$(Specimen count) of terrestrial species.

	Ordinary regression				Phylogenetic generalised least squares			
Group	Coef.	SE	t-value	p-value	Coef.	SE	t-value	p-value
Angiosperms	**-0.870**	**0.011**	**-76.51**	**< 0.001**	**0.38**	**0.025**	**15.216**	**< 0.001**
					**(± 0.013)**	**(± < 0.001)**	**(± 0.497)**	**(± < 0.001)**
Epiphytic families	**-0.742**	**0.012**	**-63.64**	**< 0.001**	**0.385**	**0.025**	**15.616**	**< 0.001**
					**(± 0.013)**	**(± < 0.001)**	**(± 0.500)**	**(± < 0.001)**
Orchidaceae	**-0.473**	**0.021**	**-22.81**	**< 0.001**	**0.286**	**0.041**	**6.968**	**< 0.001**
					**(± 0.039)**	**(± 0.001)**	**(± 0.889)**	**(± < 0.001)**
Bromeliaceae	**0.322**	**0.048**	**6.678**	**< 0.001**	**0.533**	**0.084**	**6.386**	**< 0.001**
					**(± 0.009)**	**(± 0.001)**	**(± 0.135)**	**(± < 0.001)**
Ericaceae	**-0.405**	**0.063**	**-6.466**	**< 0.001**	0.129	0.068	1.895	0.076
					(± 0.024)	(± < 0.001)	(± 0.36)	(± 0.101)
Araceae	**0.720**	**0.064**	**11.17**	**< 0.001**	**0.784**	**0.08**	**9.796**	**< 0.001**
					**(± 0.007)**	**(± < 0.001)**	**(± 0.081)**	**(± < 0.001)**
Piperaceae	**0.183**	**0.062**	**2.930**	**0.003**	**0.199**	**0.065**	**3.038**	**0.003**
					**(± 0.028)**	**(± 0.005)**	**(± 0.18)**	**(± 0.001)**
Gesneriaceae	**0.746**	**0.062**	**11.16**	**< 0.001**	**0.858**	**0.101**	**8.51**	**< 0.001**
					**(± 0.027)**	**(± 0.001)**	**(± 0.256)**	**(± < 0.001)**
Melastomataceae	0.138	0.08	1.709	0.088	**0.59**	**0.097**	**6.061**	**< 0.001**
					**(± 0.024)**	**(± 0.001)**	**(± 0.253)**	**(± < 0.001)**
Apocynaceae	**-0.617**	**0.118**	**-5.216**	**< 0.001**	**0.338**	**0.14**	**2.408**	**0.018**
					**(± 0.026)**	**(± 0.001)**	**(± 0.182)**	**(± 0.01)**
Rubiaceae	**-0.566**	**0.117**	**-4.856**	**< 0.001**	-0.338	0.15	-2.272	0.053
					(± 0.091)	(± 0.006)	(± 0.663)	(± 0.07)
Cactaceae	**0312**	**0.138**	**2.261**	**0.024**	0.269	0.23	1.173	0.257
					(± 0.059)	(± 0.007)	(± 0.273)	(± 0.096)

Cyclanthaceae	**0.454**	**0.205**	**2.217**	**0.028**	**0.575 (± 0.178)**	**0.223 (± 0.027)**	**2.529 (± 0.45)**	**0.019 (± 0.012)**
Begoniaceae	**1.408**	**0.233**	**6.052**	**< 0.001**	**1.393 (± 0.037)**	**0.235 (± 0.001)**	**5.933 (± 0.161)**	**< 0.001 (± < 0.001)**
Araliaceae	-0.366	0.377	-0.973	0.331	**0.793 (± 0.0244)**	**0.346 (± 0.003)**	**2.290 (± 0.077)**	**0.023 (± 0.005)**
Balsaminaceae	0.073	0.228	0.318	0.751	0.072 (± 0.002)	0.228 (± < 0.001)	0.318 (± 0.009)	0.751 (± 0.007)
Urticaceae	0.643	0.332	1.940	0.053	**0.752 (± 0.028)**	**0.311 (± 0.002)**	**2.418 (± 0.098)**	**0.016 (± 0.005)**
Asteraceae	**0.630**	**0.272**	**2.318**	**0.021**	0.607 (± 0.224)	0.316 (± 0.021)	1.919 (± 0.711)	0.118 (± 0.169)
Solanaceae	0.165	0.315	0.522	0.601	**0.814 (± 0.081)**	**0.365 (± 0.007)**	**2.226 (± 0.197)**	**0.029 (± 0.02)**
Crassulaceae	**0.846**	**0.301**	**2.813**	**0.005**	**1.00 (± 0.025)**	**0.292 (± 0.001)**	**3.418 (± 0.09)**	**0.001 (± < 0.001)**
Campanulaceae	**1.076**	**0.282**	**3.814**	**< 0.001**	**1.458 (± 0.064)**	**0.301 (± 0.004)**	**4.839 (± 0.213)**	**< 0.001 (± < 0.001)**
Zingiberaceae	0.400	0.269	1.474	0.141	**0.69 (± 0.028)**	**0.274 (± 0.002)**	**2.52 (± 0.108)**	**0.012 (± 0.004)**
Nepenthaceae	0.395	0.348	1.136	0.259	0.492 (± 0.101)	0.342 (± 0.007)	1.446 (± 0.324)	0.172 (± 0.09)
Lentibularaceae.	**0.889**	**0.385**	**2.309**	**0.022**	**0.881 (± 0.022)**	**0.385 (± 0.001)**	**2.286 (± 0.058)**	**0.023 (± 0.004)**
Asparagaceae	0.457	0.421	1.084	0.278	0.218 (± 0.066)	0.382 (± 0.003)	0.571 (± 0.171)	0.573 (± 0.119)
Schlegeliaceae	-0.239	0.465	-0.513	0.612	-0.239 (± < 0.001)	0.465 (± < 0.001)	-0.513 (± < 0.001)	0.612 (± < 0.001)

Table S3: Estimated effects of epiphytism on range size measured as $\log_{10}$(Extent of occurrence) according to ordinary linear regression models and phylogenetic generalised least squares regression models for angiosperms, angiosperms within families containing at least one epiphytic species and each family with more than ten epiphyte species. Coefficients show estimated difference to $\log_{10}$(Extent of occurrence) of terrestrial species.

	Ordinary regression				Phylogenetic generalised least squares			
Group	Coef.	SE	t-value	p-value	Coef.	SE	t-value	p-value
Angiosperms	**-0.411**	**0.035**	**-11.77**	**<0.001**	**0.434 (± 0.02)**	**0.068 (± <0.001)**	**6.379 (± 0.288)**	**<0.001 (± <0.001)**
Epiphytic families	**-0.210**	**0.036**	**-5.762**	**<0.001**	**0.446 (± 0.02)**	**0.070 (± 0.007)**	**6.402 (± 0.284)**	**<0.001 (± <0.001)**
Orchidaceae	**-0.392**	**0.071**	**-5.536**	**<0.001**	0.087 (± 0.073)	0.14 (± 0.003)	0.627 (± 0.525)	0.525 (± 0.25)
Bromeliaceae	**1.32**	**0.161**	**8.235**	**<0.001**	**1.284 (± 0.037)**	**0.269 (± 0.002)**	**4.765 (± 0.166)**	**<0.001 (± <0.001)**
Ericaceae	0.177	0.146	1.207	0.227	0.017 (± 0.028)	0.171 (± 0.001)	0.099 (± 0.161)	0.884 (± 0.092)
Araceae	0.073	0.163	0.448	0.654	**0.873 (± 0.018)**	**0.208 (± 0.001)**	**4.19 (± 0.084)**	**<0.001 (± <0.001)**
Piperaceae	-0.274	0.192	-1.422	0.154	-0.227 (± 0.082)	0.2 (± 0.014)	-1.167 (± 0.452)	0.289 (± 0.242)
Gesneriaceae	**0.920**	**0.200**	**4.617**	**<0.001**	**0.883 (± 0.044)**	**0.278 (± 0.004)**	**3.173 (± 0.125)**	**0.002 (± 0.001)**
Melastomataceae.	**0.504**	**0.225**	**2.244**	**0.025**	**1.342 (± 0.062)**	**0.318 (± 0.003)**	**4.221 (± 0.198)**	**<0.001 (± <0.001)**
Apocynaceae	0.007	0.547	0.012	0.99	0.512 (± 0.106)	0.633 (± 0.006)	0.809 (± 0.165)	0.425 (± 0.099)
Rubiaceae	0.015	0.362	0.040	0.968	0.184 (± 0.126)	0.392 (± 0.004)	0.469 (± 0.321)	0.652 (± 0.191)
Cactaceae	0.108	0.35	0.309	0.757	0.242	0.552	0.439	0.666

					(± 0.096)	(± 0.012)	(± 0.178)	(± 0.126)
Cyclanthaceae	-0.461	0.425	-1.084	0.280	-0.458	0.426	-1.076	0.285
					(± 0.028)	(± 0.011)	(± 0.075)	(± 0.046)
Begoniaceae	1.113	0.607	1.832	0.067	1.11	0.608	1.825	0.069
					(± 0.027)	(± 0.002)	(± 0.045)	(± 0.007)
Araliaceae	0.315	0.947	0.333	0.739	0.784	0.980	0.801	0.425
					(± 0.073)	(±0.005)	(±0.074)	(±0.042
Balsaminaceae	-0.115	0.946	-0.122	0.903	-0.098	0.945	-0.103	0.909
					(± 0.057)	(± 0.004)	(± 0.061)	(± 0.026)
Urticaceae	1.644	1.079	1.525	0.128	1.977	1.059	1.866	0.063
					(± 0.054)	(± 0.002)	(± 0.051)	(± 0.007)
Asteraceae	0.550	0.587	0.937	0.349	0.764	0.679	1.121	0.309
					(± 0.336)	(± 0.041)	(± 0.485)	(± 0.223)
Solanaceae	0.095	0.602	0.158	0.875	0.064	0.713	0.091	0.918
					(± 0.063)	(± 0.015)	(± 0.091)	(± 0.06)
Crassulaceae	-0.213	0.632	-0.337	0.736	0.24	0.612	0.393	0.696
					(± 0.058)	(± 0.003)	(± 0.095)	(± 0.07)
Campanulaceae.	-0.640	0.610	-1.048	0.295	1.23	0.659	1.863	0.086
					(± 0.264)	(± 0.014)	(± 0.393)	(± 0.098)
Zingiberaceae	0.200	0.867	0.231	0.818	0.2	0.867	0.231	0.818
					(± <0.001)	(± <0.001)	(± <0.001)	(± <0.001)
Nepenthaceae	0.016	0.997	0.016	0.987	0.016	0.997	0.016	0.987
					(± <0.001)	(± <0.001)	(± <0.001)	(± <0.001)
Lentibularaceae.	**2.164**	**1.081**	**2.003**	**0.047**	1.96	1.077	1.82	0.073
					(± 0.135)	(± 0.01)	(± 0.128)	(± 0.018)
Asparagaceae	0.727	0.953	0.762	0.446	0.065	0.922	0.07	0.934
					(± 0.094)	(± 0.002)	(± 0.102)	(± 0.071)
Schlegeliaceae	**-3.397**	**0.879**	**-3.864**	**0.001**	**-3.374**	**0.889**	**-3.811**	**0.002**
					**(± 0.119)**	**(± 0.048)**	**(± 0.265)**	**(± 0.004)**

Table S4: Estimated effects of epiphytism on range size measured as Number of botanical countries according to generalised linear models with quasipoisson error distributions for angiosperms, angiosperms within families containing at least one epiphytic species and each family with more than ten epiphyte species. Coefficients show estimated difference to number of botanical countries of terrestrial species.

	Generalised linear regression			
Group	Coef.	SE	t-value	p-value
Angiosperms	**-0.443**	**0.015**	**-28.74**	**<0.001**
Epiphytic families	**-0.311**	**0.015**	**-21.1**	**<0.001**
Orchidaceae	**-0.386**	**0.019**	**-20.49**	**<0.001**
Bromeliaceae	**0.600**	**0.047**	**12.630**	**<0.001**
Ericaceae	**-0.313**	**0.112**	**-2.807**	**0.005**
Araceae	-0.209	0.118	-1.765	0.078
Piperaceae	**0.634**	**0.056**	**11.221**	**<0.001**
Gesneriaceae	**0371**	**0.039**	**9.402**	**<0.001**
Melastomataceae	-0.135	0.079	-1.712	0.087
Apocynaceae	-0.057	0.100	-0.566	0.572
Rubiaceae	-0.028	0.129	-0.219	0.826
Cactaceae	**0.656**	**0.080**	**8.164**	**<0.001**
Cyclanthaceae	0.168	0.155	1.109	0.278
Begoniaceae	**1.141**	**0.081**	**14.16**	**<0.001**
Araliaceae	0.261	0.246	1.061	0.289
Balsaminaceae	-0.082	0.308	-0.267	0.789
Urticaceae	**0.936**	**0.209**	**4.473**	**<0.001**
Asteraceae	0.239	0.267	0.896	0.370
Solanaceae	0.075	0.302	0.249	0.804
Crassulaceae	0.074	0.290	0.256	0.798

Campanulaceae.	-0.393	0.397	-0.991	0.322
Zingiberaceae	0.344	0.189	1.815	0.070
Nepenthaceae	-0.215	0.242	-0.888	0.376
Lentibularaceae.	0.090	0.411	0.219	0.827
Asparagaceae	0.061	0.334	0.183	0.854
Schlegeliaceae	-0.157	0.323	-0.485	0.631

Table S5: Summary of GBIF download citations.

GBIF.org (21 March 2022) GBIF Occurrence Download https://doi.org/10.15468/dl.wmerwv
GBIF.org (21 March 2022) GBIF Occurrence Download https://doi.org/10.15468/dl.uj3k3y
GBIF.org (21 March 2022) GBIF Occurrence Download https://doi.org/10.15468/dl.95tcrx
GBIF.org (21 March 2022) GBIF Occurrence Download https://doi.org/10.15468/dl.t22czn
GBIF.org (23 March 2022) GBIF Occurrence Download https://doi.org/10.15468/dl.nje5yj
GBIF.org (27 March 2022) GBIF Occurrence Download https://doi.org/10.15468/dl.u9zmqc

Table S6: Number and percentage of species which occur in four or fewer botanical countries included in each of the two GBIF-derived datasets (EOO and specimen count). We chose to tabulate percentage of species per group which occur in four or fewer botanical countries rather than percentage of total number of species per group in order to better illustrate data coverage and potential biases between groups in GBIF data, given that we did not attempt to download GBIF data for the 16% of angiosperm species found in five or more botanical countries.

	Number of species in EOO analysis			Number of species in Specimen count analysis		
	All species	Epiphytes	Terrestrials	All species	Epiphytes	Terrestrials
Angiosperms	145,898<br>(52%)	8,170<br>(33%)	137,728<br>(54%)	244,453<br>(88%)	20,322<br>(83%)	224,131<br>(88%)

Orchidaceae	7,634 (29%)	4,811 (26%)	2,823 (38%)	20,900 (80%)	14,899 (80%)	6,001 (81%)
Bromeliaceae	1,719 (52%)	929 (54%)	790 (50%)	3,078 (93%)	1,614 (94%)	1,464 (92%)
Ericaceae	2,812 (68%)	531 (64%)	2,281 (69%)	3,833 (93%)	792 (95%)	3041 (92%)
Araceae	1,625 (44%)	506 (72%)	1,119 (37%)	3086 (84%)	685 (97%)	2,401 (80%)
Piperaceae	1,563 (44%)	289 (49%)	1,274 (43%)	3,214 (90%)	574 (97%)	2,640 (89%)
Gesneriaceae	1,490 (41%)	346 (59%)	1,144 (38%)	3,146 (88%)	539 (92%)	2,607 (87%)
