# Supplementary material for: Geographic range size and rarity of epiphytic flowering plants
Source: Nat Plants. 2025 Jun 13;11(7):1380–9. doi: 10.1038/s41477-025-02022-9 (PMC12283404; doi:10.1038/s41477-025-02022-9)
Supplement: Supplementary file 2 — Reporting Summary [file 41477_2025_2022_MOESM2_ESM.pdf]

nature portfolio

Corresponding author(s): Vida J. Svahnstrom

Last updated by author(s): Apr 2, 2025

Reporting Summary

Nature Portfolio wishes to improve the reproducibility of the work that we publish. This form provides structure for consistency and transparency in reporting. For further information on Nature Portfolio policies, see our Editorial Policies and the Editorial Policy Checklist.

Statistics

For all statistical analyses, confirm that the following items are present in the figure legend, table legend, main text, or Methods section.

n/a	Confirmed	
☐	☒	The exact sample size (*n*) for each experimental group/condition, given as a discrete number and unit of measurement
☐	☒	A statement on whether measurements were taken from distinct samples or whether the same sample was measured repeatedly
☐	☒	The statistical test(s) used AND whether they are one- or two-sided<br>*Only common tests should be described solely by name; describe more complex techniques in the Methods section.*
☐	☒	A description of all covariates tested
☐	☒	A description of any assumptions or corrections, such as tests of normality and adjustment for multiple comparisons
☐	☒	A full description of the statistical parameters including central tendency (e.g. means) or other basic estimates (e.g. regression coefficient) AND variation (e.g. standard deviation) or associated estimates of uncertainty (e.g. confidence intervals)
☐	☒	For null hypothesis testing, the test statistic (e.g. *F*, *t*, *r*) with confidence intervals, effect sizes, degrees of freedom and *P* value noted<br>*Give P values as exact values whenever suitable.*
☒	☐	For Bayesian analysis, information on the choice of priors and Markov chain Monte Carlo settings
☒	☐	For hierarchical and complex designs, identification of the appropriate level for tests and full reporting of outcomes
☒	☐	Estimates of effect sizes (e.g. Cohen's *d*, Pearson's *r*), indicating how they were calculated

Our web collection on statistics for biologists contains articles on many of the points above.

Software and code

Policy information about availability of computer code

Data collection	The package rgbif (v. 3.7) was used in R version 4.1.2 to collect occurrence record data from GBIF.
Data analysis	All data analysis was carried out using R version 4.1.2. Packages used in the data collection process include: rWCVP (v. 1.0.1), rCAT (v. 1.6), CoordinateCleaner (v. 2.0.20), and phylolm (v. 2.6.5). Scripts created for this analysis are available in a public GitHub repository at https://github.com/vsvahn/GlobalEpiphytes.

For manuscripts utilizing custom algorithms or software that are central to the research but not yet described in published literature, software must be made available to editors and reviewers. We strongly encourage code deposition in a community repository (e.g. GitHub). See the Nature Portfolio guidelines for submitting code & software for further information.

Data

Policy information about availability of data

All manuscripts must include a data availability statement. This statement should provide the following information, where applicable:

- Accession codes, unique identifiers, or web links for publicly available datasets
- A description of any restrictions on data availability
- For clinical datasets or third party data, please ensure that the statement adheres to our policy

The version of the World Checklist of Vascular Plants (Specia Issue version) used in our study is available at doi:10.34885/rar9-jx25. Lifeform data was obtained from the EpiList 1.0 (https://doi.org/10.1002/ecy.3326). Occurrence records were downloaded from the Global Biodiversity Information Facility (https://www.gbif.org/) –

see Supplementary Materials for GBIF download DOIs. The data used for the analysis are available at https://doi.org/10.5281/zenodo.15122031

Research involving human participants, their data, or biological material

Policy information about studies with human participants or human data. See also policy information about sex, gender (identity/presentation), and sexual orientation and race, ethnicity and racism.

Reporting on sex and gender	N/A
Reporting on race, ethnicity, or other socially relevant groupings	N/A
Population characteristics	N/A
Recruitment	N/A
Ethics oversight	N/A

Note that full information on the approval of the study protocol must also be provided in the manuscript.

Field-specific reporting

Please select the one below that is the best fit for your research. If you are not sure, read the appropriate sections before making your selection.

☐ Life sciences ☐ Behavioural & social sciences ☒ Ecological, evolutionary & environmental sciences

For a reference copy of the document with all sections, see nature.com/documents/nr-reporting-summary-flat.pdf

Ecological, evolutionary & environmental sciences study design

All studies must disclose on these points even when the disclosure is negative.

Study description	We used regressions (ordinary generalized linear regressions and phylogenetic generalized least squares regressions) to test the effect of lifeform (epiphyte vs. non-epiphyte) on range size. We ran separate regressions for three range size metrics: number of native botanical countries, extent of occurrence (EOO) derived from occurrence records, and number of specimens derived from occurrence records. For each of these three analyses (range size ~ lifeform), we ran regressions across angiosperms as well as for each angiosperm family with >10 epiphytic species.
Research sample	We used distribution data (occupied botanical countries) in the World Checklist of Vascular Plants for all angiosperm species not of hybrid origin with known distributions. We downloaded GBIF occurrence records derived from herbarium specimens for all angiosperm species occurring in 4 or fewer botanical countries for the EOO and specimen count analyses. See Methods for more details.
Sampling strategy	We calculated number of occupied botanical countries for all angiosperms and occurrence record-derived metrics (EOO and specimen count) for all angiosperms occurring in four or fewer botanical countries with specimen records data in GBIF.
Data collection	We used the 'Special Issue' version of the World Checklist of Vascular Plants to collect distribution data for all angiosperms at the level of botanical countries. We collected GBIF data in a series of downloads using the R package 'rgbif', DOIs for data downloads are provided in the supplementary materials. We obtained a lifeform variable (epiphyte/non-epiphyte) from combining lifeform data in WCVP and using a published list of epiphyte species 'Epilist 1.0' - see Methods for more details.
Timing and spatial scale	Each data download (i.e. sampling) occurred once, with dates provided in the supplementary materials.
Data exclusions	GBIF downloads only included species occurring in four or fewer botanical countries, and only included occurrences derived from herbarium specimens. These exclusions are clearly justified in Methods.
Reproducibility	Range size data used in our analyses is provided in a Zenodo repository. All code required to collect and clean the data and run the analyses is provided in a Github repository. See Data and Software and Code sections for links.
Randomization	Not relevant to our study, which did not include test subjects.
Blinding	Not relevant to our study, which did not include test subjects.

Did the study involve field work? ☐ Yes ☒ No

Reporting for specific materials, systems and methods

We require information from authors about some types of materials, experimental systems and methods used in many studies. Here, indicate whether each material, system or method listed is relevant to your study. If you are not sure if a list item applies to your research, read the appropriate section before selecting a response.

Materials & experimental systems

n/a	Involved in the study
☒	☐ Antibodies
☒	☐ Eukaryotic cell lines
☒	☐ Palaeontology and archaeology
☒	☐ Animals and other organisms
☒	☐ Clinical data
☒	☐ Dual use research of concern
☒	☐ Plants

Methods

n/a	Involved in the study
☒	☐ ChIP-seq
☒	☐ Flow cytometry
☒	☐ MRI-based neuroimaging

Plants

Seed stocks	*Report on the source of all seed stocks or other plant material used. If applicable, state the seed stock centre and catalogue number. If plant specimens were collected from the field, describe the collection location, date and sampling procedures.*
Novel plant genotypes	*Describe the methods by which all novel plant genotypes were produced. This includes those generated by transgenic approaches, gene editing, chemical/radiation-based mutagenesis and hybridization. For transgenic lines, describe the transformation method, the number of independent lines analyzed and the generation upon which experiments were performed. For gene-edited lines, describe the editor used, the endogenous sequence targeted for editing, the targeting guide RNA sequence (if applicable) and how the editor was applied.*
Authentication	*Describe any authentication procedures for each seed stock used or novel genotype generated. Describe any experiments used to assess the effect of a mutation and, where applicable, how potential secondary effects (e.g. second site T-DNA insertions, mosiacism, off-target gene editing) were examined.*
